# Editorial: Ciliates: Key Organisms in Aquatic Environments

**DOI:** 10.3389/fmicb.2022.880871

**Published:** 2022-03-29

**Authors:** Weiwei Liu, Xinpeng Fan, Jae-Ho Jung, Jean-David Grattepanche

**Affiliations:** ^1^Key Laboratory of Tropical Marine Bio-resources and Ecology, South China Sea Institute of Oceanology, Chinese Academy of Science, Guangzhou, China; ^2^School of Life Sciences, East China Normal University, Shanghai, China; ^3^Department of Biology, Gangneung-Wonju National University, Gangneung, South Korea; ^4^Department of Biology, Temple University, Philadelphia, PA, United States

**Keywords:** aquatic environment, biogeography, phylogeny, taxonomy, trophic role

Ciliates are a diverse clade of microbial eukaryotes and one of the most morphologically complex and highly differentiated taxa among single-celled organisms. These organisms exhibit a combination of unique characters, such as nuclear dimorphism (somatic macronucleus and germline micronucleus), sexual reproduction by conjugation, highly specialized organelles and complex cytoskeletal structures. These characteristics make them important model organisms for the study of cell biology, genetics and evolution. In addition, ciliates are key drivers of the biogeochemical cycling of nitrogen, carbon, and other elements, and play an essential role in energy flow in aquatic systems as the linker between bacterial and primary productions and higher trophic levels.

Despite their significance in aquatic ecosystems, our understanding of the diversity, evolution and ecological roles of ciliates is relatively limited. Over 8,000 nominal species have been reported to date, but the overwhelming majority of them are not described by modern standards (e.g., molecularly). It has been estimated that 83–89% of free-living ciliate species are not discovered, especially from under-sampled habitats such as the deep ocean and tropical marine environments (Foissner et al., 2008). Only a small proportion of ciliates have been studied phylogenetically. This is mainly due to (1) the lack of accurate and easily verifiable taxonomy data upon which to build molecular studies and (2) the difficulty to assess their dual genomes (e.g., some species have gene-size chromosomes, silent diploid micronucleus genome containing a large number of genes) (Chen et al., 2021). Therefore, phylogenetic relationships among many ciliate groups remain unclear. Furthermore, ciliates' ecophysiology and diversity can be impacted by environmental factors. However, they generally tend to be lumped together with other protist groups in ecological studies rather than being investigated as an independent group. Therefore, there is a pressing need for exploring their ecological response to environment variations at the individual, population, and community levels.

This Research Topic gathered 25 articles on ciliates across marine and freshwater environments. These studies investigated their diversity and evolutionary relationships based on morphological, morphogenesis and phylogenetic analyses, and characterize their ecological function, community organization, biogeography, and interactions with organisms at various trophic levels in aquatic habitats.

A great number of articles are devoted to the taxonomy of ciliates isolated from a variety of aquatic habitats. In total 33 morphotypes including six prostomateans (Frantal et al.; Jiang et al.), 11 hypotrichs (Gao et al.; Kumar et al.; Ma J. et al.; Omar et al.; Shao et al.; Song, Zhang, et al.; Zhang et al.; Zhu et al.), two litostomateans (Chi et al.), six peritrichs (Wang Z. et al.; Wu et al.), two heterotrichs (Jin et al.), one protocruzean (Liang et al.), four suctorians (Ma M. et al.), and one oligotrich (Tsai et al.), were described in detail from observations of live cells and silver-stained specimens. Based on the morphology, morphogenesis and molecular phylogeny, their systematic relationships were revealed. It is notable that among these 17 species and three genera, namely *Foissnerophrys* (Jiang et al.), *Wilbertophrya* (Ma J et al.), and *Pseudosincirra* (Gao et al.), were new to science, and some poorly known species such as *Peritromus kahli* Villeneuve-Brachon, 1940, *Zoothamnium arbuscula* Ehrenberg, 1831 and *Zoothamnium hentscheli* Kahl, 1935 were studied by describing the morphological characters for the first time. The high number of new taxa indicates that there is a large uncharacterized ciliate diversity, which is consistent with the conclusions of Foissner et al. (2008). Many new species were discovered in some specific habitats such as plateau puddle, heavy metals contaminated industrial outlet, deep-sea (Mariana Trench), suggesting that further investigations on such understudied environmental conditions are likely to reveal significant numbers of new taxa awaiting discovery. In addition, some cryptic species in genera *Urotricha* and *Strombidium* were identified in some studies (Frantal et al.; Tsai et al.), which highlight the value of an integrative approach including traditional taxonomic, modern molecular and ecological investigations for improved understanding of ciliate biodiversity.

Three articles studied the biogeography of ciliates. Song, Xu, et al. analyzed the regional distribution of strombidiids in China coasts based on diversity data and found that species number was higher in southern China compared to northern China. They also infered their global distribution based on SSU rRNA gene sequence data and indicated that the species isolated from Chinese coastal waters were likely to be globally distributed. The distribution patterns of ciliate diversity in the South China Sea were analyzed by Liu et al., and revealed that ciliate communities presented a clear niche preference—the community variation between the intertidal area and the neritic/oceanic areas was more significant than that between neritic and oceanic areas. In the intertidal water, the community was not significantly different among sites but did differ among habitat types. In neritic and oceanic areas, the spatial variation of communities among different sites was clearly observed. In addition, they found that environmental selection was the major process structuring the taxonomic composition in intertidal water, while spatial processes played significant roles in influencing the taxonomic composition in neritic and oceanic water. Li H. et al. investigated the tintinnid community across the North Pacific Transition Zone (NPTZ) and found that the species were divided into five groups: boreal, warm water type I, warm water type II, transition zone and cosmopolitan species. Their results confirmed the existence of tintinnid transition zone community and suggested that the warm water type I group could be used as an indicator of the northern boundary of the NPTZ.

Two articles are focused on genome analysis of ciliates. For the first time, the codon usage bias of the macronuclear genome in free-living ciliates were analyzed by Wang Y. et al.. The results showed that the GC contents in the macronuclear genomes of ciliates were lower than 50% and appeared AT-rich. The codon usage pattern and evolution of ciliates were affected by genetic mutation and natural selection. The codons of most ciliates ended with A or U and eight codons were the general optimal codons. A CRISPR/Cas9 expression vector of *Stylonychia lemnae* was constructed by optimizing the macronuclear genome codon and was successfully used to knock out the Adss gene. To better understand transcription regulation on gene-sized chromosomes, Zheng et al. generated macronuclear genome assemblies for two ciliates. They identified distinct AT-rich sequences conserved in both species, at both the 5′ and 3′ end of each gene. Their transcriptomic data showed the 5′ cis-regulatory element was associated with active gene expression. Gene family evolution analysis suggested nanochromosomes originated in spirotrichs more than 900 million years ago, and gene families that play a role in transcription factor binding and sequence-specific DNA binding rapidly expanded in these two species.

Three articles concerned the physiology of ciliates. The functionality of the sequestered chloroplasts in *Strombidium* species is thought to be independent from any nuclear control. Maselli et al. investigated the potential retention of prey genetic material in this ciliate and demonstrated that genetic material from prey nuclei, nucleomorphs and ribosomes was detectable inside the ciliate for at least 5 days after prey ingestion. Moreover, single-cell transcriptomic revealed the presence of transcripts of prey nuclear origin in the ciliate after 4 days of prey starvation. These new findings might lead to the reconsideration of the mechanisms regulating chloroplasts retention in ciliates. To understand the diet-tissue fractionation values of ciliates, Park et al. conducted experiments to determine their carbon and nitrogen isotopic fractionation values. The Δ13C and Δ15N for all ciliates represented significantly positive enrichments. Irrespective of the dietary type, both Δ13C and Δ15N were very similar for the same ciliate species. These results suggested that Δ13C and Δ15N for marine ciliates were similar to those found in other marine organisms with very little food-dependent variation. In addition, one article discussed the bionomic strategy of ciliates to persist in harsh environments. Li Y. et al. reviewed the studies on resting cysts in ciliates. They summarized the inducing factors of encystment and excystment, and presented their morphological change and molecular mechanisms during these dynamic processes, which provided new perspectives for studies on adaptive evolution of unicellular eukaryotes.

In conclusion, the collection of articles in this Research Topic not only reveals the high diversity and evolutionary relationships of ciliates, but also provides novel information on ciliates ecophysiology traits, e.g., distribution pattern, genomic regulation and interaction with prey. These impressive contributions will further close the knowledge gap about their roles in nature and boost the development of ciliatology.

## Author Contributions

WL led writing of the manuscript. All authors contributed to manuscript revision, read, and approved the submitted version.

## Funding

WL was supported by the National Natural Science Foundation of China (Grant No. 32070517) during the writing of the manuscript.

## Conflict of Interest

The authors declare that the research was conducted in the absence of any commercial or financial relationships that could be construed as a potential conflict of interest.

## Publisher's Note

All claims expressed in this article are solely those of the authors and do not necessarily represent those of their affiliated organizations, or those of the publisher, the editors and the reviewers. Any product that may be evaluated in this article, or claim that may be made by its manufacturer, is not guaranteed or endorsed by the publisher.
